# Multicenter prospective cohort study on colorectal adenoma recurrence and malignant transformation risk based on integrative medicine: a study protocol

**DOI:** 10.3389/fonc.2025.1657390

**Published:** 2025-10-27

**Authors:** Yi-Fan Hui, Shao-Chen Song, Wen-Jian Zhu, Shu Bu, Jin Sun, Ting-Sheng Ling, Hai-Bo Cheng

**Affiliations:** ^1^ The First Clinical Medical College, Nanjing University of Chinese Medicine, Nanjing, China; ^2^ Affiliated Hospital of Nanjing University of Chinese Medicine, Nanjing, China; ^3^ Jiangsu Collaborative Innovation Center of Traditional Chinese Medicine in Prevention and Treatment of Tumor, Nanjing, China

**Keywords:** colorectal adenoma, traditional Chinese medicine, integrative medicine, multicenter prospective cohort study, recurrence and malignant transformation risk

## Abstract

**Introduction:**

Colorectal adenoma (CRA), the main precursor of colorectal cancer (CRC), has a high recurrence rate (5-year cumulative: 48 - 68%) and a malignant transformation risk (0.2 - 0.5%). Traditional Chinese Medicine (TCM) provides a potential method to improve risk assessment and prevention. Based on TCM theories and the characteristics of colorectal adenoma, we hypothesize that an integrated system combining TCM syndromes, constitution types, and modern medical parameters can better predict adenoma recurrence and CRC occurrence.

**Methods and analysis:**

This is a multicenter prospective cohort study. A total of 15,000 patients with colonoscopy-confirmed colorectal adenomas will be recruited from 12 tertiary hospitals in China. At baseline, TCM characteristics (syndromes, constitution, tongue and pulse features), endoscopic findings, and biological samples (tongue coating, saliva, stool, blood, tissue) will be collected. The participants will be followed up for 5 years with risk-stratified standardized colonoscopy intervals. The primary outcomes are adenoma recurrence and CRC occurrence. The secondary outcomes include identifying risk factors and developing a predictive model. Multivariable logistic regression, Cox models, and mixed-effects models will be used for data analysis. A comprehensive biobank will be established to support future mechanistic studies.

**Ethics and dissemination:**

Ethical approval has been obtained from the Institutional Review Board of the Affiliated Hospital of Nanjing University of Chinese Medicine. Written informed consent will be obtained from all participants, who can withdraw at any time without prejudice.

**Clinical trial registration:**

https://clinicaltrials.gov/study/NCT06136026, identifier NCT06136026.

## 1 Introduction

Colorectal adenoma (CRA), characterized by abnormal proliferation of colorectal mucosal epithelium, represents the most significant precancerous lesion for colorectal cancer (CRC) (1). Global data indicate that the 5-year cumulative recurrence rate after adenoma resection reaches 48%-68%, with approximately 0.2%-0.5% progressing to malignancy (2). In China, with the widespread adoption of endoscopic screening, the detection rate of CRA has been steadily increasing (3). Both domestic and international guidelines consider that the current optimal treatment for CRA is endoscopic resection, which can significantly reduce the incidence of CRC (1–4). Expert consensus on endoscopic diagnosis and treatment for colorectal cancer and precancerous lesions in China (2023, Guangzhou) states that for colorectal adenomatous polyps or polyps with a long diameter ≥5 mm, endoscopic resection is recommended, and the follow-up interval should be comprehensively determined based on factors such as pathological nature, lesion size, and number (1). The European Society of Gastrointestinal Endoscopy (ESGE) guidelines and the National Comprehensive Cancer Network (NCCN) guidelines, on the other hand, suggest that different endoscopic treatment methods can be selected according to the different types of polyps (3, 4). While risk factors for CRA have been studied, with pathological diagnosis as the gold standard and endoscopic resection as the primary intervention, large-scale cohort studies are still needed for further validation (4). TCM has shown promise in the diagnosis and prevention of adenoma recurrence, though more evidence is needed to support its clinical efficacy and mechanisms (5).

The theory of TCM constitution and syndrome differentiation provides a unique theoretical framework for assessing overall health status. Research has demonstrated that in CRA, dampness-heat syndrome is associated with increased disease progression risk, while spleen deficiency syndrome correlates with poor postoperative recovery (6). Guidelines have identified phlegm-dampness, dampness-heat, and blood stasis constitutions as high-risk factors for adenoma development (7). Tongue and pulse diagnosis, as essential components of TCM diagnostics, offer non-invasive, convenient, and reproducible assessment methods. Studies have shown that tongue features correlate with adenoma pathology: pale tongue with tubular adenomas, purple tongue with villous adenomas, and thick white coating with tubular-villous adenomas (7).

In addition, the potential of biological sample analysis in CRA screening and prognostic evaluation has drawn increasing attention. Studies have demonstrated that the tongue-coating lipidome in CRA patients shows significant differences compared to both healthy individuals and CRC patients, with nine key lipid species (AUC = 0.9) enabling effective discrimination between CRA and CRC (8). Specific lipids may serve as biomarkers for distinguishing adenomas and identifying malignant transformation. Analyses of saliva and fecal microbiota in patients with colorectal polyps also reveal statistically significant differences in structure and diversity compared to healthy controls (9). Shifts in the relative abundance of certain genera have been validated as diagnostic biomarkers for polyps, either individually or in combination. While conventional markers such as serum CEA and CA19–9 are associated with colorectal tumor progression, they often suffer from limited specificity and high testing costs. Recently, omics-based biomarker research has paved new avenues for the prognostic evaluation of adenomas.

Current research faces three main limitations: first, the lack of standardized assessment systems for constitution classification, syndrome diagnosis, tongue/pulse feature evaluation, and biological sample collection affects result comparability and reproducibility; second, small sample sizes limit statistical power; and third, short follow-up periods preclude long-term prognosis evaluation. While some studies suggest associations between specific TCM constitutions, syndromes, and biomarkers with adenoma recurrence risk, the evidence level remains low, necessitating more rigorous prospective studies.

Based on these considerations, we have designed this multicenter, prospective cohort study to enroll 15,000 CRA patients for a 5-year follow-up, systematically evaluating the associations between TCM constitution, syndromes, tongue/pulse features, multi-omics biomarkers, and adenoma recurrence and malignant transformation risk. This cohort systematically collected the exposure of patients to risk factors related to CRA, such as dietary structure, history of smoking and alcohol consumption, family history of polyps, etc., and followed up the pathological conditions of polyps in the same patients, aiming to explore the relationship between the occurrence and carcinogenesis of CRA and related risk factors.The primary objectives are to: (1) establish a standardized integrated TCM-Western medicine adenoma case and sample database; (2) assess correlations between various exposure factors and adenoma recurrence/progression; and (3) develop an integrated prognostic prediction model incorporating TCM and Western medicine characteristics (10). The characteristics of this cohort are: (1) wide distribution of centers across various regions in China; (2) significant differences in diet and climate across regions, enabling exploration of regional variations in adenomas; (3) comprehensive collection of information including patients’ demographic data, adenoma-related risk factors, medical history, TCM symptoms and constitution, examination results, etc.; (4) instead of solely collecting patients with a specific TCM syndrome type, focusing on specific symptoms and TCM constitution of patients, allowing for interpretation of rich syndrome types. This study’s innovations include: conducting the first large-scale multidimensional prediction study, implementing standardized assessment protocols, and integrating traditional TCM diagnostics with modern biomarkers. The results will provide crucial evidence for developing precise risk assessment systems and individualized prevention strategies.

## 2 Methods and analysis

### 2.1 Trial design

This study is designed as a longitudinal, prospective, large-sample, multicenter cohort study with an integrative Chinese and Western medicine approach, focusing on patients with CRA in China. The study aims to systematically follow patients from the time of adenoma diagnosis through potential progression or recurrence stages, providing comprehensive data on the natural history, risk factors, and outcomes of CRA in the Chinese population. This integrative research model combines TCM perspectives with modern Western medical approaches to create a more comprehensive evaluation system.

### 2.2 Study population

This study will recruit 15,000 newly diagnosed CRA patients across 12 hospitals in China. As a prospective, multicenter integrative Chinese-Western medicine cohort study, we will confirm CRA diagnosis through colonoscopy and pathological verification, adhering to the ‘Consensus on Pathological Diagnosis of Gastrointestinal Adenoma and Benign Epithelial Polyps’ (11) (Digestive System Group, Chinese Pathology Society). Participating medical centers encompass diverse geographic regions across eastern, central, and western China, with patient populations including referrals from external institutions to ensure broad demographic representation. Tertiary hospitals with specialized facilities were selected as research centers, possessing substantial expertise in standardized colonoscopic procedures and pathological diagnosis to guarantee diagnostic accuracy and follow-up integrity.

Inclusion criteria: (1) meeting the diagnostic criteria for CRA; (2) age ≥18 years; (3) signed informed consent.

Exclusion criteria: (1) history of malignant tumors; (2) previous radiotherapy, chemotherapy, or colorectal surgery; (3) severe cognitive impairment, dementia, or psychiatric disorders.

### 2.3 Interventions

#### 2.3.1 Study measures and collection procedures

**Figure 1:**
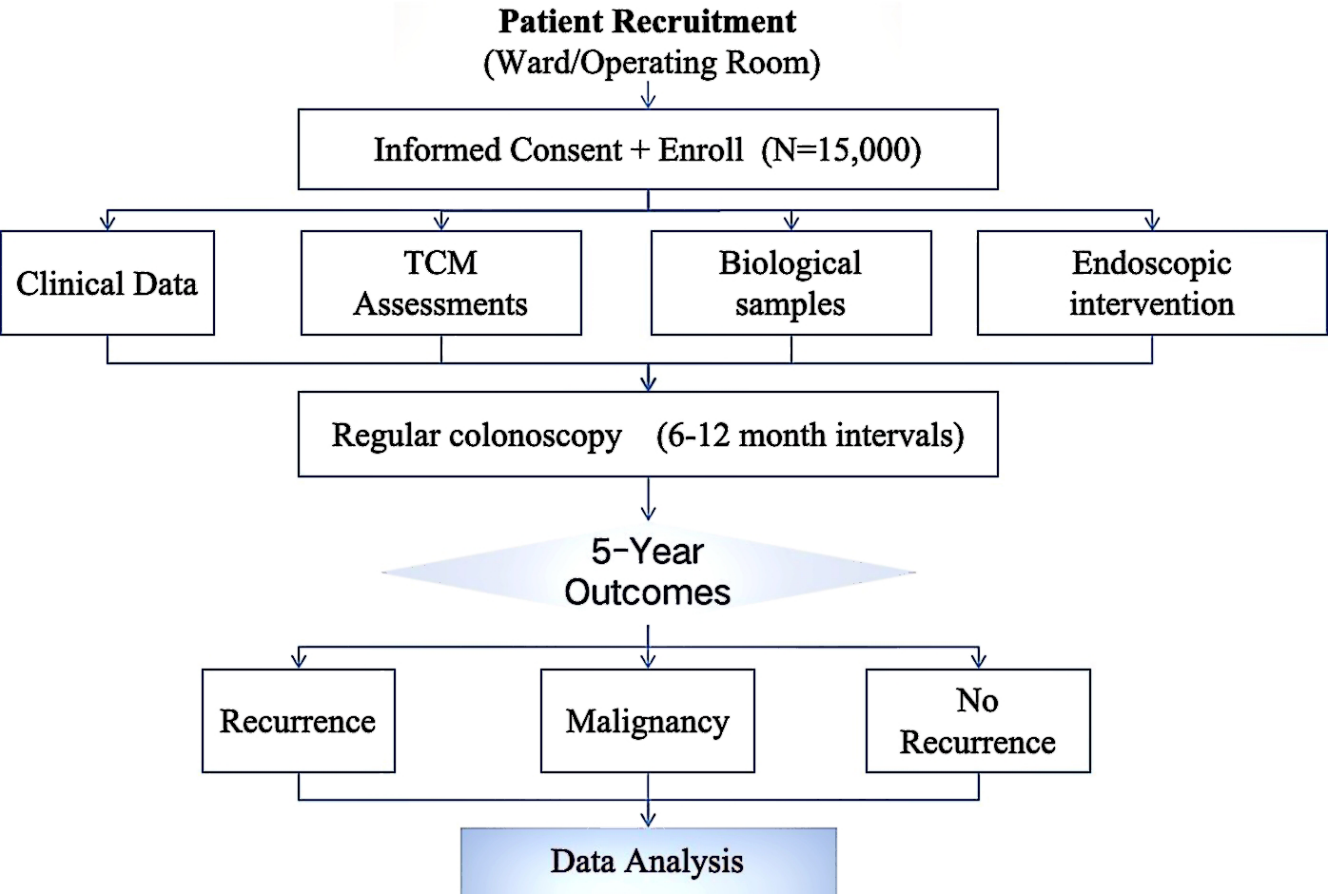
Comprehensive technical roadmap for colorectal adenoma surveillance and outcome analysis.

This prospective, multicenter cohort study of CRA in China will implement a comprehensive data collection protocol to ensure high-quality research data. All measurements will be conducted according to standardized procedures across all participating centers (**Figure 1**).

#### 2.3.2 Baseline and follow-up data collection

At baseline, demographic information including age, gender, height, weight, smoking status, alcohol consumption, dietary habits, physical activity patterns, family history, medical history, and concomitant medications will be collected through structured interviews by trained research staff. This information will provide valuable context for understanding risk factors associated with CRA development and recurrence (**Table 1**).

**Table 1:** Data collection and measurement schedule.

Time Point		Baseline	Follow-up(T1)	Follow-up(T2)	Follow-up(T3)	Follow-up(T4)	Follow-up(T5)
		-7–0 days	12 months	24 months	36 months	48 months	60 months
Demographic data	Medical history	✓	✓	✓	✓	✓	✓
	Family history	✓	–	–	–	–	–
Clinical examination	Colonoscopy findings	✓	✓	✓	✓	✓	✓
	Pathological examination	✓	✓	✓	✓	✓	✓
Laboratory tests	Complete blood count	✓	–	–	–	–	–
	Blood biochemistry	✓	–	–	–	–	–
	Fecal occult blood test	✓	–	–	–	–	–
	Tumor markers	✓	–	–	–	–	–
TCM syndrome evaluation	TCM syndrome questionnaire	✓	✓	✓	✓	✓	✓
	Tongue diagnosis	✓	✓	✓	✓	✓	✓
	Pulse diagnosis	✓	✓	✓	✓	✓	✓
Biological samples	Blood	✓	–	–	–	–	–
	Tongue coating	✓	–	–	–	–	–
	Stool	✓	–	–	–	–	–
	Saliva	✓	–	–	–	–	–
	Adenoma tissue	✓	–	–	–	–	–
Risk factor assessment	Smoking and alcohol use	✓	✓	✓	✓	✓	✓
	Dietary habits	✓	✓	✓	✓	✓	✓
	Physical activity	✓	✓	✓	✓	✓	✓
Treatment information	Western medical treatment	✓	✓	✓	✓	✓	✓
	TCM treatment	✓	✓	✓	✓	✓	✓
	Concomitant medications	✓	✓	✓	✓	✓	✓
Outcome assessment	Adenoma recurrence	–	✓	✓	✓	✓	✓
	Colorectal cancer occurence	–	✓	✓	✓	✓	✓

Demographic data, family history, tongue coating, and saliva samples are collected only at baseline, while most clinical and laboratory measurements are repeated at each follow-up visit to track changes over time and identify potential predictors of adenoma recurrence or progression.

Baseline TCM diagnostic information (tongue appearance, pulse characteristics, and symptomatic manifestations) will be collected simultaneously by trained research personnel. Collection timing will be strictly standardized to 24–48 hours post-admission and prior to colonoscopy, exclusively during morning fasting state (8-hour food restriction, 2-hour fluid restriction) to eliminate temporary interference from dietary intake or endoscopic procedures. This observational study will enroll only patients with first-time CRA detection (confirmed through comprehensive history review and colonoscopy of no previous adenoma history). At baseline, participants will receive no adenoma-specific treatments (including oral Chinese herbal medicine, herbal enemas, or other TCM interventions). During hospitalization (mean duration ≤1 week), patients will receive only routine Western medications related to surgical procedures (e.g., bowel preparation agents), ensuring baseline TCM diagnostic data accurately reflected the pathological status. Post-discharge self-administration of Chinese herbal medications is possible; this aspect will be systematically monitored during the follow-up period.

Clinical data collection will focus on comprehensive documentation of adenoma characteristics through colonoscopy and pathological examination. Colonoscopy findings will include the number, size, location, and morphological features of adenomas. Pathological assessment will document adenoma type (tubular, tubulovillous, villous), degree of dysplasia, and margin status. These parameters are essential for risk stratification of patients and determination of appropriate surveillance intervals.

Laboratory examinations encompassed complete blood count, blood biochemistry (liver and kidney function, blood glucose), fecal occult blood test, and comprehensive biomarker profiling [traditional tumor markers (CEA, CA19-9), emerging biomarkers (neutrophil-to-lymphocyte ratio (NLR), triglyceride-glucose index (TyG) (12), and serum metabolites including glycerophospholipids/sphingolipids)] (13). Additionally, multi-omics data (genomics, transcriptomics, metabolomics) from CRA patients were analyzed to identify novel biomarkers associated with adenoma recurrence and malignant transformation. Baseline sampling was conducted within 7 days prior to initial endoscopic examination, while follow-up sampling was performed 48–72 hours before surveillance endoscopy, ensuring precise correlation between biomarker fluctuations and endoscopically confirmed adenoma status.

**Figure 2:**
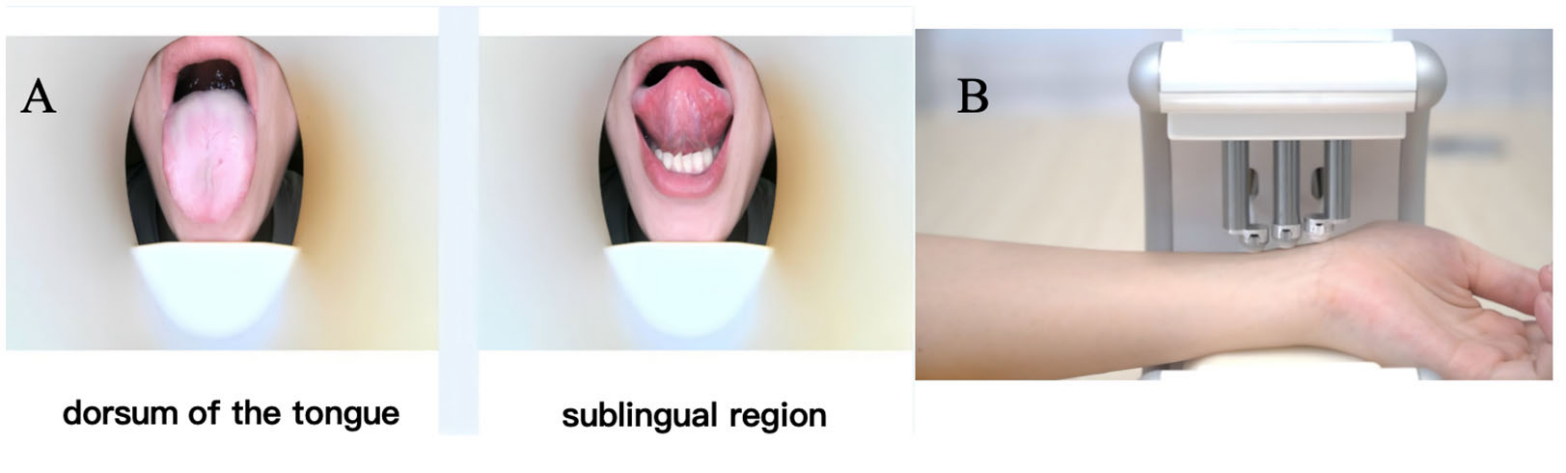
Workflow of tongue and pulse diagnostic image acquisition in TCM. **(A)** Tongue diagnosis via the standardized tongue imager from Zhejiang Cancer Hospital. **(B)** Pulse diagnosis with the standardized pulse device from Dajing TCM, plus biological sample collection for mechanism research and biomarker discovery.

An important feature of this integrative Chinese-Western medicine research is the collection of TCM syndrome data. This will include:

1. TCM syndrome questionnaires focusing on major symptoms (abnormal stool characteristics, altered bowel habits, abdominal pain, abdominal distension) and minor symptoms (gastric discomfort, fatigue, fever, chills, headache).
2. The TCM Constitution Questionnaire was developed by Professor Wang Qi from Beijing University of Chinese Medicine.
3. Tongue diagnosis using standardized tongue imaging equipment developed by Zhejiang Cancer Hospital (**Figure 2A**). Equipped with a 60-megapixel Leica lens and D65 standard light source, this device ensures accurate tongue color reproduction. Participants were required to fast and abstain from water for 4 hours before data collection to avoid interference with tongue coating color. During imaging, participants sat upright in a quiet environment, extended their tongues naturally, and remained relaxed to minimize tongue deformation. Images were captured only after confirming no obstruction of the tongue, with requirements to fully display both the tongue body and coating. RGB values were recorded, using RGB (255, 224, 189) as the reference standard. Subsequent image processing involved standardization of RGB and HSV channels to ensure color consistency, and a stable algorithm was applied to improve the accuracy of tongue diagnosis.
4. Perform pulse diagnosis using the standardized pulse diagnostic device developed by Dajing Traditional Chinese Medicine (**Figure 2B**). This device is fitted with high-sensitivity pressure sensors, which accurately contact the “Cun”, “Guan”, and “Chi” pulse positions on the wrist for data acquisition. Prior to measurement, participants fasted and refrained from water for 4 hours, maintained physical relaxation, and ensured correct wrist positioning. After the examiner confirmed the pulse points, participants placed their forearms on the device. The instrument automatically collected pulse waveforms and key parameters to ensure data reproducibility. Pulse data were uploaded to an analysis system, where an intelligent algorithm generated pulse diagnosis results. Key pulse waveform parameters included pulse amplitude and cycle, with output results classifying pulse patterns such as “Chen” (deep), “Shuo” (rapid), “Xu” (deficient), “Shi” (excessive), and "Chi"(slow).
5. Blood samples: 6-8ml of whole blood was collected using EDTA anticoagulant tubes, processed within 4 hours to separate plasma, and stored at -80 °C.
6. Tongue coating samples: collected using sterile swabs from patients in a fasting state before tooth brushing.
7. Stool samples: collected fecal swabs before patient endoscopic treatment.
8. Saliva samples: 5ml or more, collected after patients abstain from food, water, and oral hygiene for 1 hour.
9. Adenoma tissue: collected during endoscopic procedures, with paired normal mucosa when possible.

#### 2.3.3 Follow-up schedule and procedures

Follow-up examinations will be conducted at 12, 24, 36, 48, and 60 months after enrollment. During each follow-up visit:

1. Clinical examination encompassed comprehensive history-taking (with emphasis on gastrointestinal symptoms and treatment-related complications) and thorough physical examination. Follow-up assessments utilized structured questionnaires combined with medication verification to document patients’ TCM usage over the preceding 6 months, recorded in the ‘Follow-up Medication Record Form’ For patients reporting TCM use, TCM diagnostic data collection was immediately suspended, and patients were instructed to discontinue all TCM preparations for 1 week before returning for morning fasting reassessment. This protocol enabled dual verification through baseline-versus-follow-up data comparison and post-cessation sign monitoring, allowing differentiation between treatment-induced changes and genuine pathological manifestations.
2. Colonoscopy will be performed according to standard clinical practices to detect adenoma recurrence (**Table 2**). The timing and frequency may be adjusted based on baseline findings according to guidelines for adenoma surveillance (14, 15).
3. Updated laboratory tests similar to baseline will be performed to monitor changes and identify potential biomarkers of adenoma recurrence or progression.
4. TCM syndrome evaluation will be repeated to track changes in syndrome patterns and their potential association with disease progression.
5. Risk factor assessment will be updated, including changes in lifestyle factors (smoking, alcohol consumption, diet, physical activity).
6. Treatment information will be collected, documenting both Western medical interventions and TCM treatments.

**Table 2:** Follow-up intervals after endoscopic resection of colorectal polyps or adenomas.

Initial colonoscopy findings	Colonoscopy follow-up interval (Years)
No polyps	3–5
Hyperplastic polyp in rectum or sigmoid(diameter <10 mm)	2–3
1–2 tubular adenomas (diameter < 10 mm)	1–3
3–10 tubular adenomas	1–2
≥ 1 tubular adenoma (diameter ≥ 10 mm)	1–2
≥ 1 villous adenoma	1–2
Adenoma with high-grade intraepithelial neoplasia	1–2
Serrated lesion < 10 mm without intraepithelial neoplasia	2–3
Serrated lesion > 10 mm without intraepithelial neoplasia	1–2

In cases of special circumstances such as new symptoms (hematochezia, change in bowel habits, unexplained weight loss), additional examinations including earlier colonoscopy, enhanced CT, or MRI may be performed outside the scheduled follow-up timepoints, with these events recorded in detail.

#### 2.3.4 Data management and quality control

To ensure data quality, all research personnel will undergo standardized training prior to study initiation. Training will cover patient recruitment, data collection procedures, biological sample handling, and electronic data entry. Only personnel who pass the qualification examination will be authorized to participate in the research.

A two-level quality control system will be implemented:

1. Level-I quality control will be conducted by site coordinators who will review completed questionnaires, verify data accuracy against source documents, and ensure proper biological sample collection.
2. Level-II quality control will be performed by the central coordinating team through regular site visits, random sampling of 10% of data for source verification, and identification of potential data inconsistencies.

All data will be entered into a dedicated electronic data capture (EDC) system with built-in logic checks to identify potential errors or inconsistencies. The system will generate data queries that must be resolved before data can be considered final.

Biological samples will be managed through a standardized coding system that ensures patient confidentiality while maintaining traceability. All samples will be stored in a dedicated biobank at -80 °C, with limited access and regular inventory checks to ensure sample integrity.

#### 2.3.5 Outcomes

The primary outcomes of this prospective multicenter cohort study comprise two main endpoints: CRC occurence and CRA recurrence is defined as any histologically confirmed CRA detected during scheduled follow-up colonoscopy examinations at 12, 24, 36, 48, and 60 months after baseline. Each adenoma will be systematically evaluated according to standardized criteria, including anatomical location within the colorectum, maximum diameter measured by open biopsy forceps, and total number of lesions. Histological classification will follow the World Health Organization criteria.

For CRC occurence, the outcome is defined as any histologically confirmed CRC diagnosed during the follow-up period. Each case will undergo comprehensive assessment including TNM staging classification according to the American Joint Committee on Cancer (AJCC) 8th edition, detailed histological type and grade evaluation, and precise documentation of tumor location and size. To ensure data quality and consistency, all colonoscopy procedures will be performed by certified endoscopists following standardized protocols, and all pathological specimens will undergo centralized review by experienced gastrointestinal pathologists.

#### 2.3.6 Sample size

For this multicenter prospective cohort study, we calculated the sample size based on two primary outcomes: CRA recurrence and CRC occurrence. According to previous studies, the detection rate of CRA ranges from 15%-30% during 3-year follow-up and reaches 48%-68% during 5-year follow-up, while the annual incidence of CRC among adenoma patients is approximately 0.2%-0.5%.

We employed the cohort study sample size calculation formula N = (Zα/2 + Zβ)² × P(1-P)/d², where Zα/2 = 1.96 (α = 0.05, two-sided), Zβ = 1.28 (statistical power = 90%), and d represents the acceptable margin of error. Two primary outcomes guided our sample size determination: CRA recurrence and malignant transformation.

For the adenoma recurrence outcome, we considered the 5-year cumulative recurrence rate ranging from 48% to 68%, with a median of 58%. Using an absolute error of 2%, the minimum sample size calculation yielded 6,394 cases. After accounting for a 20% potential loss to follow-up, the adjusted sample size was 7,993 cases.

Regarding malignant transformation, the occurrence rate was estimated between 0.2% and 0.5%, with a median of 0.35%. We applied a relative error of 50% (0.175%), which resulted in a minimum sample size of 11,970 cases. Incorporating the 20% follow-up loss adjustment, the final target became 14,963 cases.

Considering the statistical requirements, research resources, and the need for robust analysis, we ultimately set the total enrollment target at 15,000 participants. This approach ensures adequate statistical power to detect clinically meaningful differences while providing comprehensive insights into CRA progression and cancer risk. This sample size ensures adequate statistical power to detect clinically meaningful differences in both primary outcomes while accounting for potential loss to follow-up. An interim analysis will be conducted when 50% of the target sample size is achieved.

#### 2.3.7 Data collection

All data will be collected using a standardized electronic case report form (eCRF) through a dedicated multicenter research platform. The eCRF includes demographic information, medical history, colonoscopy findings, pathological results, and follow-up data. Research coordinators at each participating center will be responsible for timely data entry within 3 working days after each visit. The platform features built-in logical verification rules to ensure data completeness and accuracy during entry.

Source documents including colonoscopy reports, pathology reports, and laboratory results will be scanned and uploaded to the system. For privacy protection, all patient identifiers will be replaced with unique study codes, and access to the database will be restricted through a hierarchical authority management system. The database is equipped with automatic backup systems and advanced firewalls to prevent data loss and unauthorized access.

Missing data will be actively tracked and documented with specific reasons. For participants lost to follow-up, at least three telephone contact attempts will be made before declaring the loss. Multiple imputation methods will be applied for handling missing data during analysis, and sensitivity analyses will be conducted to assess the impact of missing data patterns.

#### 2.3.8 Standardization of TCM assessments

##### 2.3.8.1 Training and calibration of TCM assessors

TCM assessor training comprised two components: theoretical knowledge and operational procedures. For theoretical training, assessors were familiarized with the structure and individual items of the TCM syndrome differentiation questionnaire and TCM constitution scale in the case report forms. For operational training, assessors received standardized instruction on questionnaire completion methods, with all data entered via EDC systems.

##### 2.3.8.2 Quality control procedures

A comprehensive quality control system was implemented under research monitor supervision. Monitors ensured complete and accurate completion of TCM syndrome questionnaires and constitution scales, checking for missing or erroneous entries.

1. Subject Selection: Subjects willing to participate in the survey were enrolled after receiving comprehensive explanation of the study significance and required information, ensuring informed consent was obtained.
2. Researcher-administered Assessment: Trained researchers conducted item-by-item interviews with subjects and completed the questionnaires accordingly. All assessment sessions were audio-recorded for quality assurance purposes.

##### 2.3.8.3 Centralized adjudication mechanism

A centralized adjudication system was established for both syndrome differentiation and constitution typing:

1. Syndrome Differentiation: Following the Guidelines for Integrated Traditional Chinese and Western Medicine Prevention and Treatment of Colorectal Adenoma, multiple licensed TCM practitioners received syndrome differentiation training. Syndrome determination was performed independently by pairs of practitioners in a blinded manner. Discrepancies were resolved through expert panel consensus. Inter-rater reliability for syndrome differentiation was assessed using Cohen’s kappa coefficient, with κ≥0.8 considered substantial agreement. Assessors achieving lower reliability underwent additional training until acceptable consistency was reached.
2. Constitution Typing: Assessors performed individual constitution typing according to the Classification and Determination of TCM Constitution standards and training protocols.

#### 2.3.9 Quality control

A three-tier quality control system will be established to ensure data quality:

First-tier: Research coordinators at each center will perform initial verification of data completeness and accuracy. They will review all CRFs against source documents before submission. Second-tier: Independent quality control supervisors will conduct monthly random checks of 10% of all entered data, comparing them with source documents. Any discrepancies identified will be resolved through queries to the research coordinators. Third-tier: The central study team will perform quarterly systematic reviews of the database, focusing on: (1) Data consistency across centers; (2) Protocol compliance; (3) Follow-up completeness; (4) Logical relationships between variables; (5) Outlier detection. Regular training sessions will be conducted for all research staff to standardize data collection procedures. Standard operating procedures (SOPs) will be established for each aspect of data collection and management. The study platform will generate monthly quality reports highlighting potential issues for immediate attention.

An independent data monitoring committee will oversee the overall data quality and safety. Site visits will be conducted every six months to verify source data and ensure protocol adherence. Any protocol violations or systematic errors identified will trigger immediate corrective actions and additional staff training if necessary.

#### 2.3.10 Statistical analysis

All statistical analyses will be performed using R version 4.0.0 and STATA version 16.0 software. Descriptive statistics will be presented as means ± standard deviations or medians (interquartile ranges) for continuous variables, and frequencies (percentages) for categorical variables. Incidence rates will be described using cumulative incidence and incidence density.

To enhance the biological validity of TCM classification, this study employs multi-omics approaches to validate associations between TCM syndrome differentiation and CRA. Metagenomics analysis utilizes high-throughput sequencing platforms to detect fecal 16S rRNA genes, employing bioinformatic tools for species identification and pathway analysis, with concurrent measurement of α and β diversity indices. Metabolomics analysis targets plasma and other biological specimens using liquid chromatography-mass spectrometry for non-targeted detection, while conducting targeted analysis of TCM-relevant metabolites, with data processed through specialized analytical platforms. Finally, biological network tools are applied to construct microbiome-metabolome interaction networks, exploring their associations with TCM syndrome patterns.

For inferential analysis, the Kaplan-Meier method will be used to estimate the cumulative incidence of CRA detection and cancer occurrence (16). Cox proportional hazards regression models (17) will be employed to analyze risk factors affecting outcomes, while competing risk models (18) will be utilized to evaluate interactions between different outcomes. Time-dependent Cox models will be used to assess the impact of dynamic risk factors on prognosis (19).

Subgroup analyses will be conducted by stratifying patients according to age, gender, and baseline adenoma characteristics. Interaction terms will be used to assess heterogeneity between subgroups. For sensitivity analyses, multiple imputation methods will be employed for handling missing data, and complete case analysis will be compared with intention-to-treat analysis. Result estimates will be assessed at different follow-up time points.

For continuous variables following normal distribution, independent sample t-tests will be used to compare differences between groups. For categorical variables, chi-square tests or Fisher’s exact tests will be applied as appropriate. Multivariate analysis will incorporate instrumental variables, multiple linear models, logistic regression (20), and Cox regression to explore the relationship between TCM syndromes and patient outcomes (21).

An interim analysis will be conducted when 50% of the target sample size is achieved. All statistical tests will be two-sided, with P<0.05 considered statistically significant.

Beyond determining the total sample size, we further considered the statistical power for key subgroup analyses. Based on prior literature reviews (22), the expected distribution of major TCM syndrome types in the study population is as follows: the “Dampness-Heat Accumulation” syndrome (dominant type) accounts for 28.70%, while the “Spleen Deficiency with Dampness Retention” and “Spleen Qi Deficiency” syndromes (major subgroups) account for 9.18% and 8.06%, respectively. With a total sample size of 15,000 participants, approximately 4,305 cases are expected to be classified under “Dampness-Heat Accumulation,” 1,377 under “Spleen Deficiency with Dampness Retention,” and 1,212 under “Spleen Qi Deficiency.” This sample allocation accurately matches the anticipated syndrome distribution, enabling the investigation of outcomes such as adenoma recurrence and malignant transformation, and fully meets the statistical requirements for comparative analyses among different TCM syndrome types.

#### 2.3.11 Informed consent

Written informed consent will be obtained from all participants before enrollment and collection of data and biological samples. The attending physician will explain all study-related information to potential participants at the outpatient department or inpatient ward after initial diagnosis of CRA.

If a participant is diagnosed with malignant tumors during the study, the attending physician will inform the participant within 24 hours; assist the participant in being referred to the oncology department or initiate a multi-disciplinary team (MDT) consultation; and re-obtain additional informed consent from the participant regarding whether to continue participating in the study.This process has been reviewed and approved by the ethics committees of all participating centers, and is subject to regular evaluation by the independent data monitoring committee.

## Discussion

This study aims to establish China’s first large-scale, multicenter integrated traditional Chinese and Western medicine (TCM-WM) cohort study for CRA. Through standardized data collection and biobank establishment, we explore the relationship between TCM syndromes and disease prognosis, providing evidence-based medical evidence for integrated TCM-WM treatment approaches.

CRA are crucial precancerous lesions, with approximately 85-90% of CRC developing through the adenoma-carcinoma sequence (23). Global research data indicates that the detection rate of CRA in the general population is approximately 20-30%, with increasing incidence trends (24). More importantly, the recurrence rate after endoscopic polypectomy remains high, imposing substantial health and economic burdens on patients. Although colonoscopy and polypectomy are currently the gold standard for CRC prevention, effective interventions for preventing adenoma recurrence are still lacking (25).

This study not only examines the relationship between TCM syndromes and disease prognosis but further investigates the molecular mechanisms underlying TCM therapies for CRA, providing comprehensive evidence-based medical support. Research demonstrates that Shenbaijiedu formula modulates immunosuppressive dendritic cell differentiation through the TMEM131-TNF signaling pathway, inhibiting malignant transformation of CRA, with its mechanism precisely elucidated via single-cell transcriptomic sequencing (26). Additionally, multicenter, randomized, double-blind, placebo-controlled clinical trials of Shenbai granules (containing Sophora flavescens, Hedyotis diffusa, Codonopsis pilosula, and stir-fried Atractylodes macrocephala) confirm that this formulation significantly reduces post-operative recurrence rates in CRA patients and decreases CRC risk. Various extracts from these constituent herbs exert their effects through multiple pathways, including signal transduction intervention, anti-inflammatory actions, immune regulation, and gut microbiota modulation (27).

In the field of CRA research, the damp-heat syndrome pattern demonstrates significant associations with specific molecular targets and pathways. Studies have revealed metabolic manifestations characterized by glucose-lipid metabolism dysregulation, particularly abnormal sphingolipid and LacNAc biosynthesis. At the signaling level, this syndrome correlates with activation of inflammatory and proliferative pathways, notably IL-17 and PI3K-Akt cascades (28). Immunologically, alterations in the immune microenvironment are observed, featuring aberrant CD8+ T-cell and macrophage infiltration, providing substantial theoretical foundation for comprehending the mechanistic role of damp-heat syndrome in CRA initiation and progression (29).

Our study innovatively constructs a standardized integrated TCM-WM cohort research model through: (1) establishing a standardized multi-center collaboration mechanism ensuring data quality; (2) implementing standardized syndrome collection methods, including objective measures such as tongue and pulse imaging systems; (3) developing a comprehensive biobank supporting future in-depth research; and (4) designing a 5-year follow-up plan to evaluate the relationship between TCM syndromes and prognosis.

This study presents several notable strengths. First, it demonstrates strong population representativeness by including 12 hospitals nationwide with an expected enrollment of 15,000 patients. Second, it employs standardized methods for syndrome assessment and biological sample collection. Third, a three-tier quality control system ensures data reliability. Fourth, the detailed follow-up plan enables long-term prognostic data collection. Finally, the multi-dimensional assessment combines clinical data, imaging examinations, and biological samples.

We acknowledge several limitations. First, selection bias may exist as participants are primarily recruited from tertiary hospitals. Second, despite utilizing objective measures, TCM syndrome assessment retains some subjectivity. Third, potential loss to follow-up might affect long-term prognostic evaluation. Fourth, multi-center collaboration may introduce heterogeneity in data collection.

This research will provide high-quality evidence-based medical support for TCM treatment of CRA, contributing to: (1) establishing standardized TCM syndrome diagnostic criteria for CRA; (2) guiding individualized treatment plan development; (3) predicting disease prognosis and optimizing follow-up strategies; and (4) promoting integrated TCM-WM treatment models.

Future research should focus on: (1) exploring relationships between TCM syndromes and molecular biomarkers; (2) developing artificial intelligence-based syndrome identification research; (3) conducting prospective clinical trials of TCM interventions; and (4) investigating mechanisms of TCM in preventing and treating CRA.

## Strengths and limitations of this study

➢ This is the first large-scale multicenter study integrating TCM and Western Medicine for CRA risk prediction.
➢ The research employs a comprehensive cohort of 15,000 patients with multimodal biological sampling and 5-year prospective follow-up.
➢ Advanced statistical modeling and machine learning techniques provide innovative approach to risk stratification.
➢ The single-country study design may limit global generalizability of findings.

## Data Availability

The original contributions presented in the study are included in the article/supplementary material. Further inquiries can be directed to the corresponding author.
